# Safety and Effectiveness of Liposuction Modalities in Managing Lipedema: Systematic Review and Meta-analysis

**DOI:** 10.1055/a-2334-9260

**Published:** 2024-08-06

**Authors:** Hatan Mortada, Sultan Alaqil, Imtinan Al Jabbar, Fatimah Alhubail, Nicolas Pereira, Joon Pio Hong, Feras Alshomer

**Affiliations:** 1 Division of Plastic Surgery, Department of Surgery, King Saud University Medical City, King Saud University, Riyadh, Saudi Arabia; 2 Department of Plastic Surgery & Burn Unit, King Saud Medical City, Riyadh, Saudi Arabia; 3 College of Medicine, King Saud University, Riyadh, Saudi Arabia; 4 College of Medicine, King Khalid University, Abha, Saudi Arabia; 5 College of Medicine, King Faisal University, Al Ahsa, Saudi Arabia; 6 Specialized Center in Lymphedema and Lipedema, Clínica Nea, Hospital del Trabajador, Región Metropolitana, Chile; 7 Department of Plastic and Reconstructive Surgery, University of Ulsan College of Medicine, Asan Medical Center, Seoul, Korea; 8 Division of Plastic Surgery, Department of Surgery, King Saud Bin Abdulaziz University for Health and Sciences, Riyadh, Saudi Arabia

**Keywords:** lipedema, liposuction, lipoedema, liposculpture, lipoplasty, quality of life, suction-assisted lipectomy

## Abstract

**Background** Lipedema is a chronic, incurable disorder characterized by painful fat accumulation in the extremities. While the application of liposuction in lipedema management has become increasingly popular, the safety and effectiveness of this approach remain contentious. Our systematic review and meta-analysis aimed to assess various liposuction modalities in lipedema management to verify their safety and efficacy.

**Methods** In-line with the Preferred Reporting Items for Systematic Reviews and Meta-analyses guidelines, we performed a comprehensive literature review from inception until March 2023 using the following electronic databases: CENTRAL, MEDLINE, Google Scholar, and EMBASE.

**Results** From the 562 initially identified articles, 20 met our inclusion/exclusion criteria for evaluation. Our review encompassed 14 prospective cohort studies, 3 retrospective studies, 2 case series, and 1 cross-sectional study. A meta-analysis of nine articles revealed a notable improvement in the quality of life, pain, pressure sensitivity, bruising, cosmetic impairment, heaviness, walking difficulty, and itching among lipedema patients who underwent liposuction. Although complications such as inflammation, thrombosis, seroma, hematoma, and lymphedema-related skin changes were reported, severe complications were rare. Crucially, no instances of shock, recurrence, or mortality were reported.

**Conclusion** Liposuction is a safe and beneficial therapeutic intervention for managing lipedema symptoms and enhancing quality of life. However, the impact of liposuction on secondary lymphedema remains unreported in the literature. Further high-quality, large-scale trials are necessary to assess the safety and effectiveness of different liposuction modalities. These studies will contribute valuable insights to optimize liposuction as a therapeutic option for individuals with lipedema.

**Level of Evidence** I, risk/prognostic study.

## Introduction

Lipedema is a chronic, symmetric, and incurable disorder impacting adipose tissue. 1 2 It typically manifests as a disproportionate, painful accumulation of fat in the extremities. 3 Commonly, it presents as bilateral enlargement of the lower limbs, including the buttocks, thighs, knees, and legs, while upper limbs are less frequently involved, and hands and feet are always spared. 4 The exact pathophysiology driving this unusual fat deposition is not yet fully understood 5 ; however, previous studies indicate potential roles for genetic and hormonal influences. 6 7 Lipedema primarily affects women, often related to their state of constant hormonal changes. 8 9 Often, patients with lipedema experience feelings of shame due to frequent misdiagnosis as obesity, 10 which can erode their trust in the health care system. 11 Furthermore, distressing symptoms such as disfigurement and pain can significantly impair quality of life, psychological health, and self-confidence. 12 As of now, lipedema remains incurable, prompting the development of different modalities to manage its symptoms. 13 However, the efficacy of conservative management is hotly debated, with most patients reportedly unresponsive to such treatment. 14 15 Thus, liposuction and its various modalities have recently gained traction as a potential means to manage lipedema's painful and disfiguring symptoms. 9 15 Nonetheless, the safety and effectiveness of liposuction modalities for lipedema management remain controversial, with limited evidence to support their use. As such, we structured this systematic review and meta-analysis to evaluate the safety and effectiveness of different liposuction modalities in managing lipedema. This study assesses the outcomes of liposuction interventions, such as tumescent liposuction, laser-assisted liposuction, ultrasound-assisted liposuction, and water-assisted liposuction, regarding their safety and efficacy in managing lipedema. The findings of this study could help guide clinical practice and inform the development of standardized protocols for liposuction interventions in lipedema management. Through this study, we aim to provide valuable insights into lipedema management and contribute to ongoing efforts to improve the quality of life for patients with this condition. A secondary objective of this research is to assess and compare the different techniques to discern the most effective approach for lipedema outcomes.

## Methods

### Search Strategy

This systematic review was designed following the Preferred Reporting Items for Systematic Reviews and Meta-analyses (PRISMA) guidelines. 16 A comprehensive literature search was conducted from database inception until March 2023, across CENTRAL, MEDLINE, Google Scholar, and EMBASE, without any timeframe restrictions. To ensure a comprehensive result, the search employed the following key terms: “lipedema OR lipoedema” AND “liposuction OR lipoplasty OR liposculpture OR fat removal OR adipose suction OR suction-assisted lipectomy OR fat removal” AND “complications OR outcomes OR patient-reported outcomes OR techniques.” The review has been registered with the International Prospective Register of Systematic Reviews (ID: CRD42023411664).

### Study Selection

The Rayyan collaboration platform was used for the initial screening of articles by title and abstract. Inclusion criteria encompassed (1) studies reporting on patients who underwent liposuction for lipedema; (2) case series and original articles; (3) studies involving adult patients aged 18 years and above; (4) studies reporting on outcomes relevant to the clinical questions; (5) studies written in English. Exclusion criteria included (1) non-English language studies; (2) editorials, letters, commentaries, or reviews; (3) studies not employing liposuction for managing lipedema; (4) studies reporting on outcomes not relevant to the study objectives; (5) studies with a high risk of bias or improper methods.

### Data Extraction

Data extraction was independently performed by two authors from the text, tables, and figures of the included studies using a predesigned, standardized extraction form. To ensure the reliability and accuracy of the extracted data, a second author independently reviewed the data extraction process, cross-checking all extracted data points against the original source materials to identify any discrepancies or missing information. This encompassed crucial data such as study characteristics (author, year of publication, study design, country of origin, and sample size), participant characteristics (age, sex, body mass index [BMI; kg/m ^2^ ], disease severity, and disease duration), liposuction techniques were systematically categorized by fluid instillation (dry, wet, tumescent) and suction technology (conventional, power-assisted, laser-assisted, ultrasound-assisted), specialized techniques were also noted, intervention characteristics (type of liposuction modality used, volume of aspirate, number of procedures, and duration of follow-up), and outcome measures. The latter included safety outcomes such as the incidence of adverse events, and effectiveness outcomes including pain, edema, mobility, quality of life, secondary lymphedema, necrosis, and recurrence. The type of statistical analysis used to evaluate study outcomes was also noted. When data were unclear or incomplete, corresponding authors were contacted for clarification. If missing data could not be obtained, a thorough explanation was provided concerning it and its potential impact on the reported results. Data management was handled by the first author, in consultation with the second author.

### Bias Assessment

Two authors independently used the methodological index for nonrandomized studies (MINORS) to assess the risk of bias in retrospective and prospective nonrandomized studies. 17 For case series, the methodological quality and synthesis of case series and case report assessment tool was utilized. 18 Potential bias was evaluated through funnel plots using the Egger's test.

### Statistical Analysis

All analyses were conducted using RevMan (version 5.4.1; The Nordic Cochrane Centre, The Cochrane Collaboration, 2020, Copenhagen). We extracted the means and standard deviations (SDs) of the scores for the questions evaluating improvements in quality of life from the included studies, both pre- and postliposuction. In our study's quantitative analysis, we excluded studies that reported median and interquartile range values, in certain analysis. This exclusion was due to the necessity of mean and SD values for certain statistical calculations, including standardized mean differences (SMDs). A weighted mean difference with 95% confidence intervals (CIs) was pooled using a fixed-effects model. Forest plots were created to evaluate the results of pooling. *p* -value less than 0.05 was considered significant. Heterogeneity between trials was assessed using the Higgin *I* ^2^ test according to the Cochrane Handbook.

### Quality Assessment and Level of Evidence

The quality of the 20 included articles was assessed by the authors, with 18 being nonrandomized noncomparative studies evaluated using the MINORS tool. 17 The remaining two case series articles were assessed using the methodological quality and synthesis assessment tool. 18 Two independent reviewers analyzed the risk of bias. This tool contains eight questions divided into four main domains: selection, ascertainment, causality, and reporting. Additionally, the MINORS tool, featuring eight items for noncomparative studies and scored on a scale of 0 (not reported), 1 (reported but inadequate), or 2 (reported and adequate), was used. The maximum score for noncomparative studies was 16.

## Results

### Literature Review

**Fig. 1:**
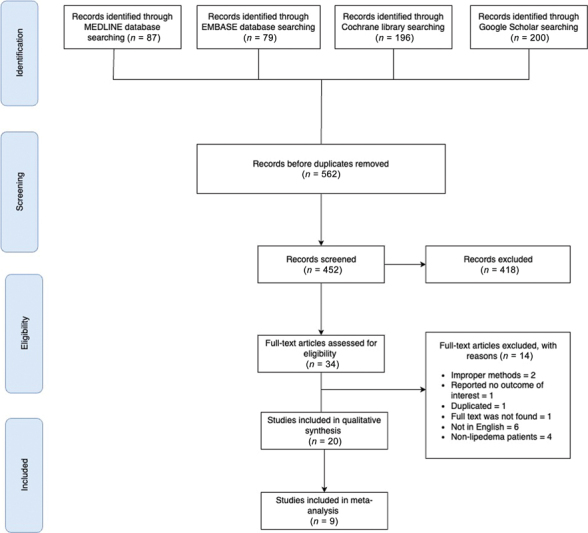
PRISMA flowchart illustrating the study selection process. The diagram outlines the number of studies identified, screened, assessed for eligibility, and included in the systematic review and meta-analysis, along with the reasons for exclusion at each stage. PRISMA, Preferred Reporting Items for Systematic Reviews and Meta-analyses.

Initially, 562 articles were sourced from various databases. After deduplication and screenings, 20 articles met the inclusion/exclusion criteria. 19 20 21 22 23 24 25 26 27 28 29 30 31 32 33 34 35 36 37 38 Fig. 1 provides an overview of the PRISMA process for conducting this systematic review. Table 1 presents the list of articles that mention the use of liposuction modalities in managing lipedema. The analysis comprised 2 case series, 1 cross-sectional study, 14 prospective cohort studies, and 3 retrospective studies. It is important to highlight that no randomized clinical trials were identified among the studies included in the analysis. Table 1 lists the articles included in our systematic review, detailing the different liposuction modalities and methods, year of publication, and country of origin.

**Table 1:** Basic demographics of the included articles

Study ID	Study design	Country	Total number of patients	Age (years)	Clinical recommendations	Level of evidence
Cornely and Gensior, 2022 19	Retrospective cohort	Germany	504	16–78	• Immediate mobilization is a part of postoperative care for lipedema patients • Combined AMLD therapy and physical treatment are recommended for 4 weeks after the procedure • A 5-day course of antibiotics is prescribed as part of postoperative care • Thrombosis prophylaxis is administered for 3 days using low-molecular-weight enoxaparin sodium	II
Kruppa et al, 2022 5	Retrospective cohort	Germany	106	18–68	• Early surgical intervention improves long-term outcomes in managing the disease • Favorable long-term outcomes are achievable in younger individuals with a body mass index of 35 kg/m ^2^ or lower • The pinch test aids surgeons in assessing the surgical outcome and determining the necessary amount of lipoaspirate	II
Wright and Herbst, 2022 21	Case series	United States	3	Case 1: 55 Case 2: 38 Case 3: 62	–	I
Baumgartner et al, 2020 22	Prospective cohort	Germany	60	22–68	• Tumescent liposuction in lipedema improves pain, edema, bruising, and movement restriction • It reduces the need for additional conservative treatments and enhances quality of life • Specialized centers with experienced surgeons should perform the procedure	I
Sandhofer et al, 2021 23	Prospective cohort	Germany	41	–	• Study participants reported a long-lasting decrease in symptom severity • The need for conservative therapy was reduced in patients even after 12 years postprocedure • The findings suggest that liposuction is an effective treatment for lipedema	II
Schlosshauer et al, 2021 24	Prospective cohort	Germany	69	24–58	• Adhering to standard guidelines for liposuction ensures the safe performance of large surgical procedures on ambulatory lipedema patients	II
van de Pas et al, 2020 25	Prospective cohort	Netherlands	117	40.9–42.2	• Tumescent liposuction treatment for lipoedema, whether under local or general anesthesia, significantly improves health-related and disease-specific quality of life • The general health status shows positive improvement with an increased number of treatment sessions	II
Witte et al, 2020 26	Prospective cohort	Germany	63	–	• Lymphatic insufficiency significantly influences the pathophysiology of lipoedema • Tumescent liposuction does not appear to reduce lymphatic function in individuals with lipoedema	II
Bauer et al, 2019 27	Cross-sectional	Germany	209	20–68	• Implementing a standardized treatment plan is essential for consistent surgical outcomes and reducing complications • Early intervention is crucial to prevent lipolymphedema and irreversible damage to the lymphatic system, emphasizing the importance of initiating treatment for lipedema at an early stage	II
Wollina and Heinig, 2019 28	Prospective cohort	Germany	111	20–81	• Early intervention with liposuction is recommended to reduce reliance on conservative treatment and prevent irreversible sequelae of lipedema, such as irreversible damage to the lymphatics.	II
Dadras et al, 2017 29	Prospective cohort	Germany	25	23–64	• Liposuction is an effective treatment for lipedema • However, it is crucial to complement liposuction with conservative measures	II
Baumgartner et al, 2016 30	Prospective cohort	Germany	85	28–75	• Liposuction is the most effective treatment for lipedema • However, to achieve maximum benefit, it is crucial to implement a comprehensive treatment concept	II
Rapprich et al, 2015 31	Prospective cohort	Germany	85	–	• Tumescent liposuction is particularly effective when applied to younger patients in the early stages of the disease, compared with older individuals with a severe form of the disease • Controlled compression therapy plays a crucial role in the overall treatment of liposuction, both before and after surgical intervention	II
Wollina et al, 2014 32	Case series	Germany	3	Case 1: 55 Case 2: 72 Case 3: 77	• Older patients with advanced disease require careful postsurgical monitoring • Common adverse events in older patients with advanced disease include temporary methemoglobinemia and leukocytosis • Although better aesthetic outcomes are typically expected in younger patients, tumescent liposuction still shows promising results in older individuals	II
Rapprich et al, 2011 33	Prospective cohort	Germany	25	22–56	• Water jet-assisted liposuction, when used with an appropriate operative technique, leads to fewer complications related to lymphatic injury • The results achieved with water jet-assisted liposuction are comparable to those of tumescent liposuction	II
Wollina et al, 2010 34	Prospective cohort	Germany	2	Case 1: 29 Case 2: 48	• Tumescent liposuction is highly effective in enhancing the quality of life for patients with lipedema • However, it is important to note that lipedema is not curable, and the use of conservative measures such as physiotherapy and compression is still necessary • Expertise is required to perform tumescent liposuction procedures safely and effectively	II
Stutz and Krahl, 2009 35	Prospective cohort	Germany	30	21–63	• Liposuction has shown a significant improvement in the quality of life for lipedema patients, including pain reduction, weight reduction, improvement in clothing size, and enhanced walking ability • However, prospective studies are needed to further evaluate and assess potential complications associated with liposuction in lipedema patients	II
Schmeller and Meier-Vollarth, 2006 36	Prospective cohort	Germany	28	22–63	• To assess the risk of postsurgical lymphatic and other complications in women with lipedema, surgeons utilizing modified suction lipectomy techniques should provide comprehensive complication reports • Longitudinal studies are required to further investigate the incidence and long-term effects of complications associated with modified suction lipectomy in lipedema patients	kIV
Schmeller et al, 2012 37	Prospective cohort	Germany	112	20–68	• Lipedema is distinct from obesity as it is not linked to metabolic disorders like type 1 or type 2 diabetes, high blood pressure, or abnormal lipid levels • Postsurgical outcomes demonstrate a noteworthy enhancement in the quality of life for lipedema patients • Further investigation is required to determine if there is a causal relationship between hypothyroidism and lipedema, addressing the potential link between the two	IV
Herbst et al, 2021 38	Retrospective cohort	United States	148	42–62	• An individualized approach to lipedema is recommended, and it proves to be effective even in cases involving multiple comorbidities and elderly patients • For older patients with multiple comorbidities, the use of prilocaine instead of lidocaine is recommended to mitigate the risk of cardiotoxicity	IV

Abbreviation: AMLD, active manual lymphatic drainage.

### Patient Profile and Basic Characteristics

The total number of patients with lipedema was reported to be 1,785. The mean age of the study participants was 39.987 years. The ages of the patients included in the studies ranged from 16 to 81 years. Among the studies that provided information on gender, a total of 1,133 participants were identified as females. Various comorbidities were identified in the included data extracted from the articles. Most commonly included hypothyroidism (75 cases), allergies (72 cases), depression (48 cases), migraine (47 cases), sleep disorders (45 cases), arterial hypertension (28 cases), and asthma and bowel disorders (27 cases) were also noted. It is important to consider potential overlaps, as individual patients may have had multiple conditions. The overall mean BMI of the included patients preintervention was 33.3 ± 5.4. One study provided data on postintervention BMI, which showed a mean of 26.1 ± 5.4, as it was 28.4 ± 4.5. 14 Additionally, another study reported a preintervention mean BMI of 35.3 and a postintervention mean BMI of 33.9. 22 Among the included studies, the most commonly reported onset trigger was puberty, documented in 65 cases. Pregnancy was identified as the trigger in 22 cases, while contraceptives and menopause were reported in 4 and 2 cases, respectively. Among the included patients, all 1,034 cases exhibited lower extremity lipedema, with 504 cases reporting involvement in the outer legs and 504 cases in the inner legs. A subset of 65 patients (6.3%) also showed upper extremity involvement in the arms. In specific cases, the affected areas were identified as arms and legs, hips and thighs, or arms, thighs, hips, knees, and calves to ankle. For the lower extremities, the most commonly affected areas were the thighs, calves, and buttocks. The thighs were further categorized into complete thighs, frontal parts, lateral sides, backside, and inside of thighs. Other affected areas included the frontal calves, calves, upper arms, forearms, back, and abdomen. In terms of leg involvement, 111 patients had lipedema, with the upper legs predominantly affected in 108 patients (97.3%) and more significant involvement in the lower legs observed in only 2 patients (1.8%). Among the 65 patients with upper extremity involvement, the arms were affected. A positive family history of lipedema was reported in the included data. About 17.44% (316 out of 1,812) of patients had a positive family history of lipedema, without specifying which family members were affected.

### Patients and Clinical Characteristics

In our analysis of the liposuction techniques across the included studies, a predominant preference for the tumescent method of fluid instillation was observed. Of the 20 studies examined, 17 (81%) used the tumescent technique. Regarding the technology used for suction, the most commonly mentioned method was power-assisted liposuction, used in 35% of the cases (7 out of 20 articles). Water-assisted liposuction was employed either solely or in combination in six studies, accounting for 29%. Tables 2 and 3 provides a summary of the liposuction techniques used in each study. In our review, 14 articles provided detailed information on the stages of lipedema in their patient cohorts. However, it is noteworthy that six articles did not specify the lipedema stages. The stage and grade distribution of lipedema among the included patients were as follows: 64 cases were classified as Stage I, 503 cases as Stage II, and 467 cases as Stage III, based on the staging system mentioned by Langendoen et al and Katzer et al. 3 6 Notably, there were no documented cases classified as Stage IV. The analysis of the data revealed that the overall mean number of treatment sessions was approximately 2.88 ± 1.30, ranging from one to five sessions per patient. The overall mean volume of aspirate removed per session was approximately 4,429.16 mL. However, it is important to note that the included studies did not consistently report the infiltrated volume, which is crucial for interpreting the volume of lipoaspirate. The duration of each liposuction session varied, ranging from 1 to 2.5 hours. Among the 20 studies included, 11 of them reported the use of compression garments postoperatively.

**Table 2:** Comparison of intervention and control groups in a study assessing liposuction modalities for lipedema

Study ID	Liposuction technique		
	Fluid instillation (dry, wet or tumescent)	Technology used for suction (conventional, power-assisted, laser-assisted, ultrasound-assisted)	Any special techniques
Cornely and Gensior, 2022 19	Tumescent	PAL	–
Kruppa et al, 2022 5	Tumescent	PAL/WAL	–
Wright and Herbst, 2022 21	NM	UAL/PAL/WAL	–
Baumgartner et al, 2020 22	NM	NM	–
Sandhofer et al, 2021 23	Tumescent	PAL	–
Schlosshauer et al, 2021 24	Tumescent	NM	Lymph-sparing liposuction
van de Pas et al, 2020 25	Tumescent	NM	–
Witte et al, 2020 26	Tumescent	WAL	–
Bauer et al, 2019 27	Tumescent	NM	–
Wollina and Heinig, 2019 28	Tumescent	Conventional/LAL	Microcannular liposuction/980-nm diode laser-assisted liposuction
Dadras et al, 2017 29	Tumescent	WAL	Vibration-assisted device
Baumgartner et al, 2016 30	NM	NM	NM
Rapprich et al, 2015 31	Tumescent	PAL/WAL	Vibration-assisted device
Wollina et al, 2014 32	Tumescent	LAL	980-nm diode laser-assisted
Rapprich et al, 2011 33	Tumescent	NM	–
Wollina et al, 2010 34	Tumescent	NM	–
Stutz and Krahl, 2009 35	Tumescent	WAL	–
Schmeller and Meier-Vollarth, 2006 36	Tumescent	PAL	–
Schmeller et al, 2012 37	Tumescent	PAL	–
Herbst et al, 2021 38	NM	NM	–

Abbreviations: LAL, laser-assisted liposuction; NM, not mentioned; PAL, power-assisted liposuction; UAL, ultrasound-assisted liposuction; WAL, water-assisted liposuction.

### Patient-reported Outcomes and Complications

**Fig. 2:**
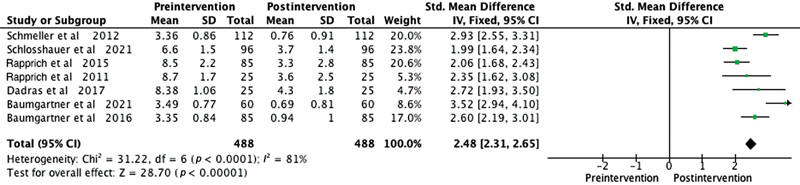
Forest plot showing the quality of life levels before and after liposuction in patients with lipedema. The standardized mean difference and corresponding 95% confidence intervals (CIs) are presented for each study. The diamond represents the overall effect size. SD, standard deviation.

**Fig. 3:**
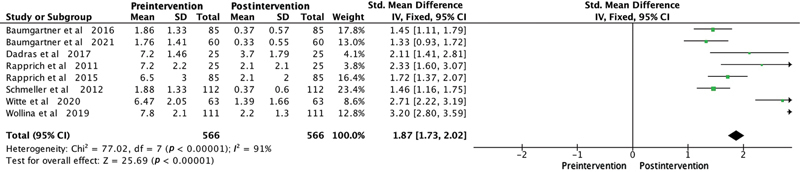
Forest plot illustrating the pain outcomes before and after liposuction in patients with lipedema. The standardized mean difference and its 95% confidence intervals (CIs) are displayed for each study. The diamond symbol represents the overall effect size. SD, standard deviation.

**Fig. 4:**
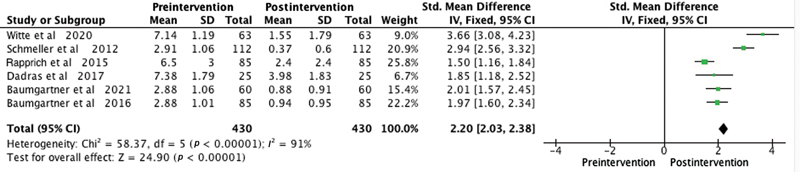
Forest plot showing sensitivity to pressure levels before and after liposuction in patients with lipedema. The standardized mean difference and corresponding 95% confidence intervals (CIs) are presented for each study. The diamond represents the overall effect size. SD, standard deviation.

**Fig. 5:**
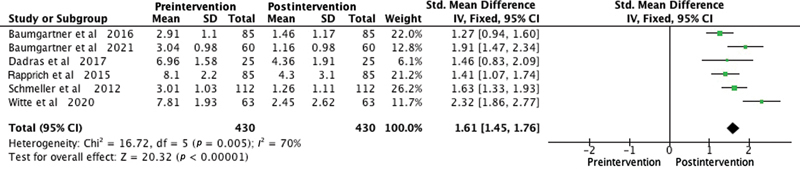
Forest plot showing bruising levels before and after liposuction in patients with lipedema. The standardized mean difference and corresponding 95% confidence intervals (CIs) are presented for each study. The diamond represents the overall effect size. SD, standard deviation.

**Fig. 6:**
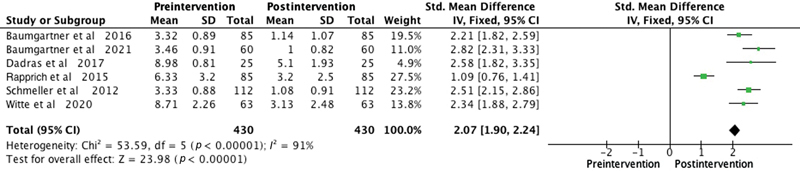
Forest plot showing cosmetic impairment levels before and after liposuction in patients with lipedema. The standardized mean difference and corresponding 95% confidence intervals (CIs) are presented for each study. The diamond represents the overall effect size. SD, standard deviation.

**Fig. 7:**
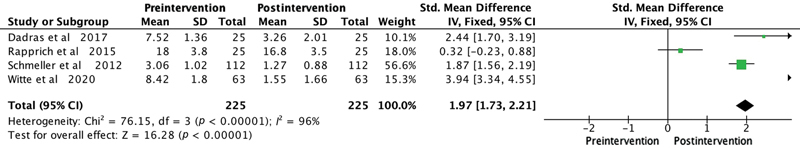
Forest plot showing the heaviness before and after liposuction in patients with lipedema. The standardized mean difference and corresponding 95% confidence intervals (CIs) are presented for each study. The diamond represents the overall effect size. SD, standard deviation.

**Fig. 8:**
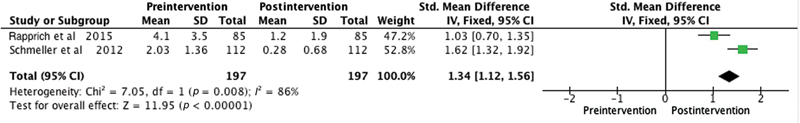
Forest plot showing the difficulty in walking levels before and after liposuction in patients with lipedema. The standardized mean difference and corresponding 95% confidence intervals (CIs) are presented for each study. The diamond represents the overall effect size. SD, standard deviation.

Among the patient satisfaction evaluation methods used in the studies, the Visual Analogue Scale was employed in six studies. One study utilized the Freiburg Life Quality Assessment for lymphatic diseases questionnaire, another used the Hanse-Klinik-approved questionnaire, and one employed the Body Shape Questionnaire/Lower Extremity Functional Scale. To overcome outcome measurement differences among the studies, we used the SMD as a summary statistic in our meta-analysis. This enabled us to compare the effects of the intervention on a consistent scale, despite variations in outcome measurement approaches. The meta-analysis consistently illustrated significant improvements postliposuction in patients with lipedema in areas like quality of life (SMD 2.48, *p* -value <0.0001; Fig. 2 ), pain (SMD 2.04, *p* -value <0.0001; Fig. 3 ), pressure sensitivity (SMD 2.2, *p* < 0.0001; Fig. 4 ), bruising (SMD 1.61, *p* -value < 0.0001; Fig. 5 ), cosmetic appearance (SMD 2.07, *p* -value <0.0001; Fig. 6 ), and heaviness (SMD 2.01, *p* -value <0.0001; Fig. 7 ). Lastly, the improvement in difficulty in walking after liposuction was reported in only two studies, showing a significant effect with a *p* -value <0.00001, SMD = 1.34 (95% CI: 1.12–1.56), and *I* ^2^ = 86% ( Fig. 8 ). We compared pre- and postliposuction data, revealing notable improvements in lipedema symptoms following the procedure. Pain levels decreased by 72.39%, sensitivity to pressure by 68.13%, bruising by 52.32%, cosmetic impairment by 57.36%, and the sensation of heaviness by 50.85%. Additionally, there was a significant reduction in difficulty in walking, which decreased by 78.47%. Overall, the reported complications included inflammation in 25 cases and thrombosis in 1 case. Individual cases presented specific complications such as skin changes consistent with lymphedema, foot and ankle swelling, dermal fibrosis, dermal sclerosis, hyperkeratosis, and persistent pigment irregularities. Other reported complications included mild arm-vein phlebitis in two patients, an episode of postsurgical anemia requiring a blood transfusion in one patient, and a microscopic pulmonary fat embolism in another patient. Some cases did not report complications, while one case reported deep vein thrombosis. Among the patients included in the study, a total of 14 individuals developed seroma (0.82%), 10 experienced infections (0.59%), 12 had hematoma (0.71%), 2 encountered bleeding (0.12%), 2 had skin necrosis (0.12%), and 3 developed secondary lymphedema (0.18%). The mean follow-up duration for the patients was 15.14 months, ranging from 1 to 96 months (8 years).

**Table 3:** Overview of liposuction techniques in included studies

Study ID	Number of treatment sessions, SD	Volume of fat removed per sessions (mL), SD	Use of compression garments	Injection technique and protocol	Follow-up duration (months)
Cornely and Gensior, 2022 19	3 procedures at intervals of no less than 4 weeks	–	• Modified CDT involves accentuated manual lymphatic drainage with compression and physical treatment • Postoperatively, the treatment lasts for 4 weeks • Initially, four AMLD sessions are performed per week, gradually reducing to one session per week	• Tumescent local anesthesia is commonly administered with analgesia or general anesthesia • Power-assisted liposuction employs a motor-driven suction system to aid in the suction process • To facilitate proper drainage, incisions made during the procedure are intentionally left open without suturing	NM
Kruppa et al, 2022 5	3 (2–3)	6,355 ± 2,797	Yes	• General anesthesia was used with 24-hour postoperative monitoring • Power-assisted or water jet-assisted liposuction techniques were employed, using a tumescent solution of saline and epinephrine, up to 6,000 mL per session. The surgical goal often involved megaliposuction, targeting approximately 6% of body weight in fat removal • Intraoperative “pinch test” determined the amount of lipoaspirate; postsurgery, a single dose of antibiotic prophylaxis was administered and hemoglobin and serum electrolytes were checked on the first day	20
Wright and Herbst, 2022 21	1	Case 1: 6,000 mL in one session Case 3: first session (arms, calves to ankles = 6,000 mL) second session (inner and anterior thighs, hips, and knees = 7,200 mL) 3rd session (knees, lower posterior thighs, and ankles = 1,800 mL)	Yes	• **Case 1:** Underwent ultrasound-assisted liposuction with 6 L of aspirate removed from arms and legs under general anesthesia • **Case 2:** Treated with power-assisted liposuction on hips and thighs under general anesthesia • **Case 3:** Received three water-assisted liposuction surgeries, totaling 13,050 mL of aspirate removal, with focus on arms, calves, thighs, hips, and knees	Case 1: 12 Case 2 and 3: 6
Baumgartner et al, 2020 22	NM	NM	Yes	NM	4, 8, and 12 years
Sandhofer et al, 2021 23	–	5,585	No	**Tumescent fluid injection technique** • Freshly prepared tumescent fluid heated to 37 °C was used • Two people simultaneously introduced the fluid under pressure using a KMI Surgical Infusion/Irrigation Pump • Infiltration cannulas were wiped toward the upper layers until the tissue became firm, indicating tumescence • “Vivomed infiltration needles 1.2 × 100 mm” were used **Liposuction technique** • The PAL liposuction system from MicroAire was utilized • Cannulas with a diameter of 3 to 4 mm were inserted through small incisions • Attention was given to the position and course of lymphatic vessels • Minimal secondary infiltration was performed if the patient experienced pain using a blunt, 40-cm long infiltration cannula with a diameter of 2 mm	For 4, 8, 12, 16, 20, 28, and 44 hours after the procedure
Schlosshauer et al, 2021 24	2.9 ± 1.9	1,868.0 ± 885.5 per side	No	NM	6 months on 20 patients only out of 69
van de Pas et al, 2020 25	NM	NM	No	• **Lymphoscintigraphy technique:** Subcutaneous injection of 99mTc in the first web space, followed by sequential imaging over feet, knees, and inguinal regions. Used mean clearance percentages of radioactive protein and inguinal uptake percentages at 2 hours postinjection as functional parameters. Abnormal clearance defined as <30% (with <20% considered abnormal and 20–30% questionable), and disturbed inguinal uptake as <10% (with <5% abnormal and 5–10% questionable) • **Tumescent liposuction protocol:** Performed according to standard treatment by Klein, executed by an experienced professional specializing in lipoedema treatment for over 15 years	
Witte et al, 2020 26	3 (1–4)	12,922 ± 2922 over the course of all operations	Preop: 60 Postop: 20	• Infiltration volume varied depending on the specific body area: 200–400 mL for the lower legs, 400–700 mL for the upper legs, and 200–300 mL for the upper limbs • The infiltration process had an approximate duration of 10 minutes	21.5
Bauer et al, 2019 27	3 ± 2	10,100 ± 9,600	Yes Preop: 163 Postop: 80	NM	12
Wollina and Heinig, 2019 28		4,700 ± 7,579	No	• Liposuction was performed using 2–3 mm blunt cannulas connected to a vacuum pump, generating a negative pressure of 686 mm Hg • General anesthesia was not utilized during the procedure • After the liposuction, the small 5-mm incisions were closed using polyamide sutures	2.0 ± 2.1, with follow-up duration between 5 and 7 years for 18 patients
Dadras et al, 2017 29	3	3,106	Yes	Tumescent liposuction was performed using a solution of saline with epinephrine (1:1,000,000) following the patient's consent	First postoperative follow-up: 16 Second postoperative follow-up: 37
Baumgartner et al, 2016 30	NM	NM	No	NM	48 and 96 months (4 and 8 years)
Rapprich et al, 2015 31	2.61 ± 1	NM	Yes (postoperative for 3–7 weeks)	• Sattler's method was used to infiltrate the tumescence solution • A continuously operating roller pump system aided in the infiltration process • Aspiration was performed using a blunt 4-mm-thick vibrating microcannula with three blunt openings	6 months
Wollina et al, 2014 32	5	4,000–6,000	Yes, postoperatively for 6 months	• Liposuction was performed using a 980-nm diode laser integrated into the cannula • Cannulas with diameters of 3 to 5 mm were utilized for the procedure • The cannulas were applied longitudinally, with smaller cannulas used for finer sculpting at the end	24–48 months (2–4 years)
Rapprich et al, 2011 33	2.5 ± 1.1	1,909 ± 874	Yes	• Vibrating cannulas with a 4-mm diameter and a handpiece attached (VibraSat®, Möller Medical, Fulda) were used • Aspiration was conducted using vibrating cannulas with three blunt openings at the tip arranged in a Mercedes star shape	6 months
Wollina et al, 2010 34	2	Case 1: 3,600 Case 2: 1,800	Yes, postsurgically for 6 months	• Liposuction was performed using blunt cannulas ranging from 2 to 5 mm in diameter • The cannulas were applied longitudinally during the procedure • Smaller cannulas were employed toward the end of the procedure for finer sculpting	Case 1: 6 Case 2: 48
Stutz and Krahl, 2009 35	NM	1,115 ± 554	No	• Infiltration was performed in all cases using a body-jet infiltration cannula (diameter = 3.5 mm) at Range 2 until sufficient anesthesia was achieved with the infiltration solution • The aspiration procedure commenced immediately without waiting for fluid infiltration	NM
Schmeller and Meier-Vollarth, 2006 36	NM	3,017	No	• All liposuction procedures were conducted under local tumescent anesthesia • The administration of intramuscular Demerol (35–100 mg), Vistaril (25 mg), and Versed (5 mg) preceded the procedure • The amount of aspirate was limited to less than 5 L • Tumescent anesthesia included 1 L of normal saline solution, 1 mL of 1:1,000 epinephrine, 50 or 75 mL of 1% lidocaine, and 12.5 mL of 8.4% sodium bicarbonate • The procedures utilized either Xomed or MicroAire power cannulas • Initially, accelerator and Mercedes-type cannulas ranging from 3.0 to 4.0 mm were used to treat all areas • Final contouring was accomplished using cannulas ranging from 2.0 to 2.5 mm	12.2 (1–26) months
Schmeller et al, 2012 37	NM	3,077	No	• Liposuction was performed on the legs, hips, and arms of each patient • Pure tumescent local anesthesia was administered for the procedure • Blunt vibrating microcannulas with diameters of 3 and 4 mm were used • The liposuction technique employed was power-assisted liposuction	NM
Herbst et al, 2021 38	2.4 ± 1.3	NM	No	NM	NM

Abbreviations: AMLD, accentuated manual lymphatic drainage; CDT, complex decongestive therapy; LAL, laser-assisted liposuction; NM, not mentioned; PAL, power-assisted liposuction; SD, standard deviation; TL, tumescent liposuction; TLA, tumescent local anesthesia; UAL, ultrasound-assisted liposuction; WAL, water-assisted liposuction.

### Quality Assessment and Bias Evaluation

The included studies in this analysis had varying levels of evidence. Out of the total studies, 15 were classified as Level II evidence. The MINORS tool was employed to gauge the quality of the nonrandomized studies included in this systematic review. 1 Total scores varied from 6 to 14, averaging 10. Items scoring the least included unbiased assessment of the study endpoint (a score of 0 in all studies), prospective calculation of the study size (a score of 0 in most studies), and a less than 5% loss to follow-up (a score of 0 in over half of the studies). Items with the highest scores were endpoints appropriate to the study's aim (a score of 2 in nearly all studies), clearly stated study aims (a score of 2 in most studies), and a follow-up period appropriate to the study's aim (a score of 2 in most studies; Table 4 ). The methodological quality and synthesis assessment tools were used to evaluate the risk of bias in case studies. 2 Both included case series scored 10.62 in terms of quality ( Table 5 ).

**Table 4:** Methodological index for nonrandomized studies assessment tool for nonrandomized noncomparative studies ( *N* = 18)

Item	Cornely and Gensior, 2022 19	Kruppa et al, 2022 5	Baumgartner et al, 2020 22	Sandhofer et al, 2021 23	Schlosshauer et al, 2021 24	van de Pas et al, 2020 25	Witte et al, 2020 26	Bauer et al, 2019 27
A clearly stated aim	1	2	2	2	1	2	2	2
Inclusion of consecutive patients	2	2	0	0	2	2	2	2
Prospective collection of data	0	0	2	2	0	2	2	0
Endpoints appropriate to the aim of the study	2	2	2	2	2	2	2	2
Unbiased assessment of the study endpoint	0	0	0	0	0	0	0	0
Follow-up period appropriate to the aim of the study	2	2	2	2	2	2	2	2
Loss to follow-up less than 5%	2	2	0	0	2	2	0	2
Prospective calculation of the study size	0	0	2	2	0	2	2	0
Total score	9	10	11	10	9	14	12	10

**Table:** 

Item	Wollina and Heinig, 2019 28	Dadras et al, 2017 29	Baumgartner et al, 2016 30	Rapprich et al, 2015 31	Rapprich et al, 2011 33	Wollina et al, 2010 34	Stutz and Krahl, 2009 35	Schmeller and Meier-Vollarth, 2006 36	Schmeller et al, 2012 37	Herbst et al, 2021 38
A clearly stated aim	2	2	2	2	2	2	2	2	2	2
Inclusion of consecutive patients	2	2	2	0	1	1	2	2	2	2
Prospective collection of data	2	2	2	2	2	2	2	0	2	2
Endpoints appropriate to the aim of the study	2	2	2	2	2	2	2	1	2	2
Unbiased assessment of the study endpoint	0	0	0	0	0	0	0	0	0	0
Follow-up period appropriate to the aim of the study	2	2	2	2	2	2	0	1	2	0
Loss to follow-up less than 5%	2	1	1	0	1	0	0	0	0	0
Prospective calculation of the study size	2	0	0	0	0	0	0	0	0	0
Total score	14	11	11	8	10	9	8	6	10	8

The items are scored 0 (not reported), 1 (reported but inadequate), or 2 (reported and adequate). The global ideal score being 16 for noncomparative studies.

## Discussion

Complex decongestive therapy is typically the initial treatment choice for lipedema in many countries. The aim of this treatment is to stop the progression of the condition and alleviate swelling. However, many patients continue to experience an increase in subcutaneous fat and a worsening of symptoms. 37 The tumescent technique for liposuction, introduced in the late 1980s, improved safety and minimized damage to lymphatic vessels. 35 36 37 38 39 40 41 Consequently, liposuction started to be considered a potential approach for treating lipedema and reducing fat tissue.

This systematic review evaluated the effectiveness and safety of liposuction in individuals diagnosed with lipedema. A total of 20 articles, encompassing 1,785 patients, were included in the review. Among these, 1,133 patients were identified as females, with no males reported in the data extracted from the articles. The majority of these patients were classified as stage 2 (503 individuals), followed by stage 3 (467 individuals), and a smaller subgroup of stage 1 (64 individuals). The most frequently identified comorbidities were hypothyroidism and allergies, followed by depression, migraine, sleep disorders, arterial hypertension, asthma, and bowel disorders. Lipedema was predominantly observed in the outer and inner legs, and arms. Tumescent liposuction was the most commonly used technique, followed by Power-assisted liposuction and Water-assisted liposuction.

The meta-analysis of nine articles showed a significant improvement in patients' overall well-being, indicating considerable positive outcomes. Liposuction was found to effectively alleviate a range of symptoms associated with lipedema, such as pain, sensitivity to pressure, bruising, cosmetic concerns, heaviness, and mobility difficulties. Additionally, the procedure provided relief from itchiness, a specific symptom experienced by patients. These findings are consistent with reviews conducted by Peprah and MacDougall 42 and Kruppa et al, 5 further supporting liposuction as an effective treatment for improving symptoms and overall quality of life in individuals with lipedema. Despite a few reported complications including inflammation, thrombosis, seroma, hematoma, and lymphedema-related skin changes, severe complications were rare. Notably, no instances of shock, recurrence, or death were reported in the analyzed cases, underscoring the overall safety of the procedure. Other studies 5 42 43 corroborate these findings, reinforcing the safety of liposuction as an intervention.

The average follow-up period for patients included in the studies was approximately 15 months, with a range from 1 to 96 months (8 years), which adds to the credibility and applicability of the findings. These results strongly advocate for liposuction as a safe, effective treatment option for managing lipedema symptoms, significantly improving in patients' overall well-being.

To the best of our knowledge, this is the first systematic review and meta-analysis to evaluate the safety and efficacy of different liposuction modalities in managing lipedema. The study has several strengths, such as compliance with the PRISMA guidelines, strict inclusion and exclusion criteria, comprehensive literature review without specific time constraints, inclusion of studies with moderate to high levels of evidence, and providing ample data to support a meta-analysis. According to the MINORS assessment tool, most studies scored a mean of 9.7 for potential bias. The methodological quality and synthesis assessment tool showed a quality score of 7, and a moderate risk of bias for the two included case series. However, the study does have limitations. First, most of our results were based on prospective cohort studies, potentially leading to some publication bias. Second, some studies did not include all the necessary details in their reported data, which may have resulted in some deficits in comparison. Third, 14 out of the 20 studies included in the review were from Germany, suggesting an underrepresentation of other areas of practice. Further research should prioritize the need for randomized control trials to assess the safety and effectiveness of different liposuction modalities. This should be accomplished through high-quality, large-scale, and multicenter studies. While there are multiple liposuction techniques that may yield similar outcomes, the lack of comparative studies hinders any definitive conclusion about the superiority of one method over another. The tumescent technique is often regarded as highly efficient with the lowest complication rates. However, the meta-analysis highlighted significant heterogeneity in the techniques utilized across studies, making direct comparisons between water-assisted, ultrasound-assisted, and power-assisted liposuction challenging. The variable reporting on the use of tumescence and its potential implications, especially in secondary lymphedema, further muddies the waters. There is a pressing need for standardized liposuction protocols and clarity on tumescence's role. Specific recommendations for standardization could include defining the composition of the tumescent solution, establishing guidelines for infiltration volume and rate, setting precise timing and duration for tumescence, and refining patient selection criteria. Additionally, outlining surgical techniques, postoperative care strategies, and outcome measurement protocols could significantly enhance the efficacy and safety of liposuction procedures. It is imperative that future research must concentrate on these areas to discern the efficacy and safety of the various liposuction techniques. Our study's limitation includes the potential overlap of patient cohorts in longitudinal studies, such as those by Schmeller et al (2012), 37 Baumgartner et al (2016, 2021), 22 30 and Rapprich et al (2011, 2015). 31 33 This overlap could lead to some patients being counted multiple times in our reported total of 1,785. Such repeated inclusions may slightly overestimate the number of unique patients, a factor to consider when interpreting our findings on lipedema prevalence and treatment outcomes. Our study highlights the importance of preoperative imaging for assessing lymphatic dysfunction in liposuction patients. We recommend future research to explore the use of imaging techniques like lymphoscintigraphy or near-infrared fluorescence imaging in preoperative evaluations. Such investigations could reveal crucial insights into lymphatic involvement, influencing surgical strategies and improving patient outcomes in lipedema and related conditions. In addition, our analysis reveals a need for more research on postoperative care in liposuction, particularly regarding the use and impact of compression garments. The inconsistency in their usage across studies suggests a lack of standardized practice. Future studies should focus on the efficacy of compression garments and their role in patient recovery. This could inform standardized guidelines for postliposuction care, optimizing patient outcomes and minimizing postoperative complications.

### Conclusion

Liposuction, especially the tumescent technique, is effective in treating lipedema, enhancing outcomes across different modalities. However, the literature lacks data on liposuction's impact on secondary lymphedema. Future research should focus on comprehensive trials with diverse designs, including long-term follow-up and cost-effectiveness studies, to evaluate the safety and effectiveness of liposuction in lipedema. Future work should also determine safe lipoaspirate volumes to minimize complications, furthering our understanding of liposuction's benefits for lipedema patients. Integral to this future research is the exploration of true lymph-preserving liposuction, guided by indocyanine green lymphangiography and the avoidance of lymphatics during the procedure, a promising approach that warrants further investigation.

**Table 5:** The methodological quality and synthesis of case series and case reports assessment tool ( *N* = 2)

Domain for evaluating the methodological quality of case reports and case series										
	Selection	Ascertainment		Causality		Reporting				
	Leading explanatory questions									
Reference	Q. 1	Q. 2	Q. 3	Q. 4	Q. 5	Q. 6	Q. 7	Q. 8	Quality score	Risk of bias
Wright et al, 2022 21	Yes	Yes	No	No	No	No	Yes	Yes	Fair quality study (7)	Moderate risk
Wollina et al, 2014 32	Yes	Yes	Yes	No	No	No	Yes	Yes	Fair quality study (7)	Moderate risk

**Selection:** (Question 1). Does the patient(s) represent(s) the whole experience of the investigator (center) or is the selection method unclear to the extent that other patients with similar presentations may not have been reported?

**Ascertainment:** (Question 2). Was the exposure adequately ascertained? (Question 3). Was the outcome adequately ascertained?

**Causality:** (Question 4). Were other alternative causes that may explain the observation ruled out? (Question 5). Was there a challenge/rechallenge phenomenon? (Question 6). Was there a dose–response effect? (Question 7). Was follow-up long enough for outcomes to occur?

**Reporting:** (Question 8). Is the case(s) described with sufficient details to allow other investigators to replicate the research or to allow practitioners to make inferences related to their own practice?
